# Mental Health Indicators for the Brazilian Psychosocial Care Network: A proposal

**DOI:** 10.1590/1518-8345.5618.3533

**Published:** 2022-08-01

**Authors:** Inacia Bezerra de Lima, Domingos Alves, Antônia Regina Ferreira Furegato

**Affiliations:** 1 Universidade de São Paulo, Escola de Enfermagem de Ribeirão Preto, Centro Colaborador da OPAS/OMS para o Desenvolvimento da Pesquisa em Enfermagem, Ribeirão Preto, SP, Brasil.; 2 Bolsista da Coordenação de Aperfeiçoamento de Pessoal de Nível Superior (CAPES), Brasil.; 3 Universidade de São Paulo, Faculdade de Medicina de Ribeirão Preto, Ribeirão Preto, SP, Brasil.

**Keywords:** Health Status Indicators, Healthcare Quality Indicator, Public Health Administration, Mental Health Services, Health Planning Guidelines, Health Facility Administration, Indicadores de Estado de Saúde, Indicador de Qualidade em Saúde, Administração em Saúde Pública, Serviços de Saúde Mental, Diretrizes para o Planejamento em Saúde, Administração de Estabelecimentos de Saúde, Indicadores de Estado de Salud, Indicador de Calidad de la Salud, Administración en Salud Pública, Servicios de Salud Mental, Diretrices para la Planificación en Salud, Administración de Establecimientos de Salud

## Abstract

**Objective:**

to propose Mental Health Indicators aimed at management of the Mental Health Care Network, starting with convergence of their use, in countries with public health organization.

**Method:**

an exploratory analysis of the indicators adopted and used in these countries, from the detailed analysis of their respective normative documents, considering the World Health Organization guidelines. After selection of the indicators, the Mental Health Matrix was adopted as a suggestion for their development and application in the Brazilian Psychosocial Care Network. The matrix was prepared in two dimensions, respecting the inclusion and exclusion criteria for the indicators studied, as follows: geographical (national/regional, local, individual), and time (entry, process and results).

**Results:**

the analysis indicates 41 indicators that presented diverse evidence regarding their use. All were allocated in the Mental Health Matrix, contributing as a metric to analyze the purpose of the Mental Health services, in the levels and phases of each dimension.

**Conclusion:**

the indicators selected, distributed in the different Mental Health Matrix dimensions, are being made available for their use in management and in the clinical practice, as well as for scientific studies and, in the future, to be used as definers of Mental Health policies.

## Resumo

**Objetivo:**

propor indicadores de saúde mental destinados a gestão da Rede de Atenção em saúde mental, a começar da convergência da sua utilização, em países com organização pública de saúde.

**Método:**

análise exploratória dos indicadores, adotados e utilizados nesses países, a partir da análise detalhada dos seus respectivos documentos normativos, considerando as orientações da Organização Mundial de Saúde. Após a seleção dos indicadores, adotou-se a Matriz de Saúde Mental como sugestão para seu desenvolvimento e aplicação na Rede de Atenção Psicossocial brasileira. Respeitando os critérios de inclusão e exclusão dos indicadores estudados, a matriz foi construída, em duas dimensões: geográfica: (nacional/regional, local, individual) e temporal (entrada, processo e resultados).

**Resultados:**

a análise aponta 41 indicadores que apresentaram evidências quanto ao seu uso. Todos foram posicionados na Matriz de Saúde Mental, contribuindo como uma métrica para analisar a finalidade dos serviços de saúde mental, nos níveis e fases de cada dimensão.

**Conclusão:**

os indicadores selecionados, distribuídos nas diferentes dimensões da Matriz de Saúde Mental, estão sendo disponibilizados para uso, para a gestão e na prática clínica, bem como para estudos científicos e, num horizonte futuro, para uso como definidor de políticas de saúde mental.

## Resumen

**Objetivo:**

proponer indicadores de salud mental para la gestión de la Red de Atención en Salud Mental, a partir de la convergencia de uso en países con organización pública de salud.

**Método:**

análisis exploratorio de los indicadores que adoptan y utilizan estos países, a partir del análisis detallado de sus respectivos documentos normativos, considerando las directrices de la Organización Mundial de la Salud. Después de seleccionar los indicadores, se sugirió adoptar la Matriz de Salud Mental para desarrollarlos y aplicarlos en la Red Brasileña de Atención Psicosocial. Respetando los criterios de inclusión y exclusión de los indicadores estudiados, la matriz fue construida en dos dimensiones: geográfica (nacional/regional, local, individual) y temporal (entrada, proceso y resultados).

**Resultados:**

el análisis indica que 41 indicadores presentaron evidencia de uso. Todos fueron posicionados en la Matriz de Salud Mental, y contribuyeron como métrica para analizar la finalidad de los servicios de salud mental, en los niveles y fases de cada dimensión.

**Conclusión:**

los indicadores seleccionados, distribuidos en diferentes dimensiones de la Matriz de Salud Mental, están disponibles para ser utilizados tanto en la gestión y en la práctica clínica, como en estudios científicos y, en un horizonte futuro, para definir políticas de salud mental.

**Highlights**

(1) Analysis of 41 indicators that presented diverse evidence regarding their use.

(2) They were distributed in the different Mental Health Matrix dimensions.

(3) Nine entry phase indicators.

(4) Fourteen process phase indicators.

(5) Eighteen result phase indicators.

## Introduction

According to the World Health Organization (WHO), people with mental disorders have disproportionately high disability and mortality rates. Individuals with major depression or schizophrenia have from 40% to 60% more chances of premature death than the general population, due to physical comorbidities that are not investigated in different situations (tumors, diabetes, cardiovascular diseases, HIV infection)^1^. Worldwide, suicide is the second most frequent cause of death among young people. Based on these facts, the WHO proposed a set of actions and goals for the member countries to adapt to their needs, seeking to change these rates. Thus, the WHO proposal emphasizes that leadership, governance and efficacy in Mental Health (MH) actions are to be strengthened, providing encompassing, adapted and responsive MH and social services at all care levels, strengthened by the information systems and by the results of research studies in this area^2^. 

For the WHO, one of the principles of these technologies is how the indicators can significantly synthesize the information for a particular phenomenon, portray a situation and, therefore, be used to evaluate an established and current situation or propose some change. The overall intentions established by the WHO provide the basis for assessing the Member States’ collective actions and achievements in relation to the global goals, but should not prevent establishing more ambitious national purposes^2^. 

A recent review study, pointing to the global panorama on the use of Mental Health Indicators (MHIs), analyzed 22 articles that presented the countries’ attempts to select or implement MHIs. However, the results shown evidence different uses of these indicators to improve policies, management and services. However, some countries are still committed to the process of discussions, survey and collection of fundamental indicators. Some other initiatives from other countries were in an incomplete implementation process or in the implementation of pilot projects, highlighting that these indicators’ capability is still unexplored. It is noteworthy that in low- and middle-income countries, the research studies on MHIs were conducted with many objections, due to the absence of essential MH services, financial conditions, legislation and/or political disposition, or even lack of guidelines for MH data management and integration^3^ ^-^ ^7^.

A study carried out in 2016 sought to identify MHIs to assess effective treatment coverage in MH. The Delphi study method was applied in two rounds (with 93 specialists from different countries, mainly middle- and low-income, such as: Ethiopia, India, Nepal, Nigeria, South Africa and Uganda). They initiated selection with a set of 876 indicators, finishing with 15 well-ranked. The study provided data on how the MH service and financial coverage can be assessed in low- and middle-income countries^4^.

In the Netherlands in 2013, a research study sought to develop a set of performance indicators that were executable, expressive and pertinent to assess the quality of the MH public system in Amsterdam. The study was initiated with 330 indicators, reaching the end with 56 indicators selected, based on an international questionnaire and presented to the parties participating in the process^5^. Another similar study was carried out in Germany. The researchers sought to describe the development of quality indicators for a quality-proof procedure for adult patients diagnosed with schizophrenia and schizotypal disorders with delusions^7^.

In Brazil, Ordinance 3,088 of 12/23/2011 created the Psychosocial Care Network (*Rede de Atenção Psicossocial*, RAPS) for people with mental alterations or disorders and with demand arising from the use of crack, alcohol and other substances, establishing a group of activities and actions in the Unified Health System (*Sistema Único de Saúde*, SUS)^8^. The following is evidenced as general purposes of this network: articulation and aggregation of the points of care of the health networks in the towns, evaluating care through welcoming, constant follow-up and emphasis on urgencies^8^. 

The SUS scope includes the RAPS, comprised as follows^8^: Primary Health Care (Basic Health Units); Specialized Psychosocial Care (*Centros de Atenção Psicossocial*, CAPS); Urgency and Emergency Care (24-hour Emergency Care Units, *Unidades de Pronto Atendimento*, UPAs); Residential Care of a transitory nature (collection units); and Hospital Care (psychiatric clinics and hospitals). With this, the RAPS is particularly complex, given that the therapeutic actions occur incorporated into the network with extensive communication with other heterogeneous and articulated systems. These actions are based on the principle of integrality, one of the basic procedures of the SUS, avoiding fragmentation and losses^9^ ^-^ ^10^. 

Through the Guidelines Notebook of the Brazilian Ministry of Health, in 2014, we only found one indicator for MH submitted by the Ministry of Health, in which it only proposed the national/regional coverage level of the CAPS, by population. Despite being relevant, it was not capable of evaluating the care practices offered in the different activities, or of estimating the efficiency of the service provided. Recently, this indicator has been discontinued by the Ministry of Health^8^. 

In the Interfederative Agreement document, for the 2017-2021 period, a new indicator was proposed for Systematic Matrix Support actions for the CAPS, with the professionals working in Primary Care, through Resolution No. 8. The proposal aimed at integrating MH care in Primary Care into the format of the international guidelines for the reorganization of the health systems^11^.

It should be noted that in 2011, Ordinance No. 1,654 of July 19^th^, composed the National Program to Improve Access and Quality of Primary Care (*Programa Nacional de Melhoria do Acesso e da Qualidade da Atenção Básica*, PMAQ-AB), notoriously intended for the Family Health Strategy (FHS). In the MH agenda, instructional and preventive actions are estimated, referring to the use of alcohol and other substances. In this ordinance, four indicators for MH were established, to be monitored monthly, namely: 1 - Proportion of MH appointments, except for alcohol and drug users; 2 - Proportion of alcohol user appointments; 3 - Proportion of drug user appointments; and 4 - Prevalence rate of alcoholism^12^.

Despite the important changes that took place in the structure of Brazilian MH organization in the last decades, a recent study^10^ showed how MH is discussed in the Brazilian and international evaluation policies, through the manifestations presented in national (Ministry of Health) and international (World Health Organization) normative documents released in August 2015. In this study, the absence of official evaluation instruments for Brazil and the incipience of documents from the different countries analyzed are highlighted, where information has become one of the procedures used by the WHO to evaluate MH quality, through the MH Atlas and the Action Plan^9^ ^-^ ^10^.

Given these facts and the absence of official MHIs in Brazil, the objective of this article is to propose Mental Health Indicators aimed at management of the Mental Health Care Network, starting with convergence of their use in countries with public health organization.

## Method

### Study design

This paper consists of a study of the MHI Exploratory Survey type. This method is aimed at knowledge discovery, without the purpose of evaluating or validating pre-established hypotheses^13^. 

Analysis of the results was performed according to the procedures established by the Evidence-Based Practice (EBP), referring to the use of MHIs. 

In EBP, it is crucial to provide diverse scientific information on programs and policies that are decisive in health care promotion, in order to produce evaluation research studies and constitute diverse evidences. The intention is to modify science in practices, mapping diverse information on evidence-based interventions from peer-elaborated literature for the context of a specific real environment^14^.

### The concept of “indicator”

The word “indicator” comes from Latin *indicare*, which means to discover, point, announce and estimate. Following the criteria of the semantic knowledge of words, proximity to the words “measure”, “inform” and “indicators” or their successors “measurement” and “information” stands out^15^ ^-^ ^16^. 

The indicators allow performing diagnoses, as well as monitoring and evaluating the individuals and management of the services. Thus, they encompass the search for clinical goals, the quality of professional and managerial care and the results obtained, as well as they assist in taking decisive actions, contributing to the improvement of the processes as a whole^17^.

Elaboration of an indicator is a complex activity, which can range from basic and direct counting of cases of a specific disease to calculation of more elaborate proportions, ratios, rates or indices, such as life perspective at birth^18^.

### Mental Health Indicator

In the Action Plan for the 2014-2020 period, the WHO points out the need to develop a basic set of MHIs, in addition to providing guidance, training and technical support aimed at developing surveillance/information systems to obtain the main MHIs and, with this, optimize use of this data to monitor health inequalities and results, as well as extend the information collected by the WHO Global MH Observatory (as a follow-up to the WHO Global Health Observatory); therefore setting up a database to monitor the global MH condition (including the advances regarding the goals determined in the Action Plan)^2^. 

### Scenario

The data were collected from normative documents and official websites of countries with public health systems, namely: Australia, England and Canada. The study scenario is justified by the absence of MHIs for the Brazilian RAPS. Given these facts, the proposal presents a set of indicators, distributed in the Mental Health Matrix (MHM) administrative dimensions.

### Period

The information was collected during the period encompassing 2018, 2019 and 2020.

### Selection criteria for the countries

The essential inclusion criterion for the 3 countries was based on confirming that they had universal public health systems, as they were similar to the Brazilian system and maintain provision of an MH network for management. This separation was limited to the English, Portuguese and Spanish languages.

There was flexibility of access to the practice of using the MHIs, in databases or normative documents.

### Instruments used to collect the information

After selecting the countries, analysis and selection of the indicators that best referred to the RAPS was initiated, always comparing them with the normative documents.

The first step was carried out with a meticulous search of the national (Ministry of Health) and international (World Health Organization, PAHO) normative documents, taken as a reference for comparison in Figure1 below.

**Figure 1:** Normative documents referring to the MHIs

Regions	Information collection source
WHO (General)	1. Mental Health Atlas^19^.; http://apps.who.int/iris/bitstream/10665/178879/1/9789241565011_eng.pdf?ua=1&ua=1; 2. 2013 - 2020 Mental Health Action Plan^2^.; http://apps.who.int/iris/bitstream/10665/89966/1/9789241506021_eng.pdf?ua=1; 3. Mental health systems in low- and middle-income countries: A transnational WHO-AIMS^*^ analysis^20^.; http://apps.who.int/iris/bitstream/10665/44151/1/9789241547741_eng.pdf
Europe	1. Minimum Dataset of the European Mental Health Indicators (European Commission)^21^.; https://ec.europa.eu/health/ph_projects/1998/monitoring/fp_monitoring_1998_annexe2_09_en.pdf; 2. 2013 - 2020 Mental Health Action Plan for Europe^22^.; http://www.euro.who.int/data/assets/pdf_file/0020/280604/WHO-Europe-Mental-Health-Acion-Plan-2013-2020.pdf
Latin America	1. Mental Health Atlas for the Americas^23^.; http://iris.paho.org/xmlui/bitstream/handle/123456789/28451/9789275119006_eng.pdf?sequence=1&isAllowed=y; 2. Reference Framework for the Implementation of the Regional Mental Health Strategy^24^.; http://iris.paho.org/xmlui/bitstream/handle/123456789/2790/Marco%20de%20Referencia%20para%20la%20Implementacion%20de%20la%20Estrategia%20en%20Salud%20Mental.pdf?sequence=5&isAllowed=y; 3. WHO-AIMS^*^ Mental Health Report - America and the Caribbean^25^.; http://www.paho.org/hq/index.php?option=com_docman&task=doc_view&gid=21325&Itemid=/&lang=en
Brazil	1. WHO-AIMS^*^ Mental Health Report - Brazil^26^.; http://www.who.int/mental_health/evidence/who_aims_report_brazil.pdf; 2. Interagency Health Information Network (*Rede Interagencial de Informação para a Saúde*, RIPSA). Basic indicators for health in Brazil: Concepts and applications^18^.; http://www.ripsa.org.br/2014/10/30/indicadores-basicos-para-a-saude-no-brasil-conceitos-e-aplicacoes-livro-2a-edicao-2008-2/; 3. Health Indicators^8^ - SUS^†^; http://www2.datasus.gov.br/DATASUS/index.php?area=0201; 4. Technical Note of the Regional, State and National Indicators from the 2013-2015 list of Guidelines, Objectives, Goals and Indicators^27^.; http://tabnet.datasus.gov.br/cgi/pacto/2015/Nota_Tecnica_Indicadores_Regionais.pdf; 5. Brazilian Federal Accounting Court. Program Evaluation Report - Mental Health Care Actions - 2005^28^.; https://www.tjmt.jus.br/intranet.arq/cms/grupopaginas/105/988/Relat%C3%B3rio_TCU_Sa%C3%BAde_Mental_2005.pdf; 6. Brazilian Federal Accounting Court. Program Evaluation Report - Mental Health Care Actions - 2010^29^.; https://portal.tcu.gov.br/lumis/portal/file/fileDownload.jsp?fileId=8A8182A14D7BBDF2014D8BB881DE0FE1; 7. PMAQ^‡^ Ordinance^12^.

* WHO-AIMS = World Health Organization Assessment Instrument for Mental Health Systems; ^†^SUS =*Sistema* Único *de Saúde* (Unified Health System); ^‡^PMAQ =*Programa Nacional de Melhoria do Acesso e da Qualidade da Atenção Básica* (National Program to Improve Access and Quality of Primary Care)

In the second phase, a set of documents was researched in governmental websites of the health systems from some countries (figure 2), in order to select those with identification of the indicators effectively used in MH management, which provided diverse information on how these indicators are used in each analysis dimension^30^. 

**Figure 2:** Normative documents referring to the MHIs

Countries	Information collection source
New Zealand	1. Health Indicators for New Zealanders with Intellectual Disability^31^.; https://www.health.govt.nz/system/files/documents/publications/health-indicators-nzders-intellectual-disability.pdf
Portugal	1. Portugal Mental Health in numbers - 2014^32^.; https://www.sns.gov.pt/wp-content/uploads/2017/08/RelAvPNSM2017.pdf; 2. 2017 National Mental Health Program^33^.; https://www.sns.gov.pt/noticias/2016/05/18/programas-de-saude-prioritarios/;
Spain	1. Mental Health Strategy of the National Health System^34^.; https://consaludmental.org/la-confederacion/; 2. Indicators for the evaluation of Mental Health systems in Spain^35^.; http://www.sepsiq.org/file/Noticias/GClin-SEPIndicadores.pdf
Chile	1. WHO-AIMS^*^ Mental Health Report - Chile^36^.; https://www.who.int/eportuguese/publications/Integracao_saude_mental_cuidados_primarios.pdf?ua=1
Canada	1. Canada Mental Health Indicators (Manual)^37^.; https://www.aihw.gov.au/getmedia/f9bb1a07-a43b-458a-9b73-64ef19d8aedd/Key-Performance-Indicators-for-Australian-Public-Mental-Health-Services-Third-Edition.pdf.aspx
United Kingdom	2. Digital Portal for the NHS Indicators^†^ ^38^.; https://fingertips.phe.org.uk
Australia	3. Mental Health services in Australia^39^.; https://www.aihw.gov.au/reports/mental-health-services/mental-health-services-in-australia/mental-health-indicators/key-performance-indicators-for-australian-public-m

* WHO-AIMS = World Health Organization Assessment Instrument for Mental Health Systems; ^†^NHS = National Health Service

Both investigation stages of the normative documents took place considering the selection/exclusion criteria of each country and its MHIs^30^.

### Selection/Exclusion criteria for the indicators

1. Not being part of the Brazilian situation (immigrants, ethnicities, etc.).
2. Social assistance indicators (found in the MHIs).
3. Previously included in another indicator.
4. Indicators that require information regarding self-assessment/application of an individual questionnaire.

### Selection criteria for the domains of the indicators (Entry - Process - Results)^40^ ^-^ ^41^

#### Data treatment and analysis

Following the steps established for this study, the phase for the *Identification of the selected MHI set* was reached, establishing the primary characteristics for each indicator. The format determined by the Interagency Health Information Network (RIPSA)^8^ ^,^ ^18^ was resorted to for this purpose, seeking the following items: Definition, Conceptualization, Source, Calculation Method and Category.

The MHM^40^ was defined as a fundamental reference, as a heuristic guide in the addition and applicability of the MHIs, at different network management levels. 3 fundamental and inseparable axes are pointed out in this matrix example, namely: ethical conception; evidence-based practices; and accumulation of experiences^41^. 

The MHM also refers to a model that can be used to increase the clinical and management benefits. This model suggests two dimensions: a geographical one, divided into three levels: national/regional, local and individual (patient) and a time one, defined by three phases: entry, process and results^40^. 

Health policies stand out at the national/regional level; however, at the local level we find the services operating in a coverage area and in the latter (individual), the user of the service itself. The following can be identified in each phase of the matrix, for example: the resources applied (entry phase), the professional actions performed in the provision of care (process phase), and the expected results at the different levels in relation to the change in the morbidity and mortality rates and in the individual levels, as well as in the population (results phase). 

The indicators referring to the results phase become more arduous to define and collect; in some circumstances, they can be confused with entry or process indicators. This is an example of the reduction in hospitalization time, categorized as a process indicator, which may constitute a results indicator at the individual level^41^.

With this model, an important task of this study was to systematize the MHIs, according to the MHM specifications, its levels and phases, taking into account the ethical issues, the scientific cues and the analysis of the experience acquired as foundations of the MH system^40^.

### Ethical aspects

The study herein presented followed the ethical requirements set forth by Brazilian Resolution CNS 466/12 and its complementary items and was approved by the Ethics Committee of one of the collaborating institutions (Protocol CAAE-93710218 10000 5393, CEP-EERP/USP No. 205/2018 of 08/24/2018).

## Results

The main results of this study were achieved based on the criteria described in the method. Thus, three countries were initially selected, in which 164 MHIs were identified. 

Applying the criterion of pertinence to the Brazilian RAPS, a set of 41 indicators was reached: 11 from Australia, 20 from England and 10 from Canada, respectively^37^ ^-^ ^39^. 

It is worth mentioning that 1,028 MHIs were found in the initial analysis, mentioned in the normative documents consulted, namely: WHO - 4 indicators, proposals for goals and actions^2^; European Commission - 36 indicators^21^; *World Health Organization Assessment Instrument for Mental Health Systems* (WHO-AIMS) for the Americas and the Caribbean - 155 indicators^25^; Pan American Health Organization - Proposals for goals and actions^23^; New Zealand - 3 indicators^31^; Portugal - Proposal for goals and actions^32^; Spain - Proposal and analysis of the selection of 661 indicators^35^; Australia - 15 indicators^39^; Canada - 55 indicators^37^; England - 95 MHIs^38^; and Brazil - 4 indicators from the National Program to Improve Access and Quality of Primary Care (PMAq)^12^.

After this process for the selection of countries with indicators pertinent to the Brazilian RAPS, knowledge extraction was carried out, where the respective reports, scientific articles and all relevant information were analyzed in the official pages of Canada, Australia and England, in order to arrive at the most effective selection of indicators. In the knowledge extraction phase, diverse information was sought on the use of indicators, calculation method, analysis and practical evidence, through consultations in https:// drive.google.com/file/d/1PTg2MdCu7HRIEM7OARvrEu00GEA8yAOQ/view?usp=sharing

It is noted that the indicators selected were confronted with the proposals of the WHO normative documents and of those presented in Figure 1.

Concomitantly with the analysis of the documents, this group of indicators was analyzed collaboratively (voluntarily) by 5 professionals from different areas. An additional consultation and analysis of all the indicators was also made by a specialist in the area of MH services and teaching (a Psychiatrist), seeking adequacy of this set of indicators for the Brazilian RAPS. Also for this selection process, criteria were established that allowed for a more specific selection for inclusion and exclusion of indicators.

*Indicators Excluded* - Of the indicators initially selected for analysis, 123 were excluded for not meeting the criteria specified in the method. A detailed breakdown of these exclusion criteria is included below:

1. 9 indicators excluded = 5.48% of 164 for representing specific interests of some countries (immigrants, ethnicities, etc.).
2. 69 (42.07%) were excluded for being part of indicators related to the Social Assistance Indicator that is already included in the MHI set).
3. 14 (8.53%) were excluded for being found in more than 1 country.
4. 31 (18.90%) were excluded because they need information regarding self-assessment/application of an individual questionnaire.

From selection of the MHI set (properly stratified in relation to their contents and uses)^42^, we started allocating them in the MHM^40^, according to their geographical dimension levels (national/regional, local and individual) and to the time dimension phases (entry, process and results). 

By adhering to this heuristic, each indicator started to be associated as a metric to evaluate the purpose of the MH services, at the levels and phases of each dimension: geographical and time.

*Time Dimension: Entry Phase (A)*

Six indicators from those selected were allocated in the National (or Regional) Level of Entry Phase 1A axis, serving as measures for evaluating the MH services at this level. The contents proposed for this phase refer to the governmental guidelines and policies, MH laws, costs for MH services and budget relocation, organization of MH contingent and employee training, and protocols and guidelines for treatments and referrals, as shown in Figure 3.

**Figure 3:** Allocation of the MHIs: Entry Phase

Geographical Dimension	Time Dimension
	A) Entry Phase
(1) National/Regional Level	1A; 1. Mental Health promotion in the school at the elementary level.; 2. Recovery program accreditation.; 3. Promotion, prevention and training in Mental Health First-Aid.; 4. Incidence of depression recorded: %^*^ of participants over 18 years old.; 5. Proportion of expenditure by level of compliance with the national standards.; 6. Mean daily bed cost.
(2) Local Level; (Take-in area)	2A; 1. New cases of psychosis: estimated incidence rate per 100,000 inhabitants^†^ aged from 16 to 64 years old.; 2. Mean cost *per* day of treatment.
(3) Individual Level	3A; 1. Depression incidence record: %^*^ of the practice recording individuals over 18 years old.

Source: Adapted^41^; ^*^% = Percentage; ^†^100,000 inhabitants = Data from the Brazilian Institute of Geography and Statistics^43^

Two indicators were allocated in the Entry Phase of the Local Level, 2A. As an evaluation metric, these indicators should be understood as a “lens”, making it possible to more clearly see the application and effectiveness of the MH laws and policies in force in the country. Organization of this level depends on whether the services are organized in health districts or sectors, or even by population coverage area. The relevant point of this level is verifying the need for the services to be locally organized (close to people’s homes), in order to offer care in the community^40^. As previously pointed out, this entry phase at the local level determines the budget of the local service and the balance of the hospital and community services, assessment of the needs of the local population, sizing of the clinical staff and combinations of clinical and non-clinical services, as well as work relationships among services. 

One indicator was allocated in the Entry Phase of the Individual Level, 3A. The indicators in this position serve as measures of the care offered directly to patients with mental disorders, their family members and their close social contacts. The MH services that were evaluated from these indicators are generally understood as the health professional’s exclusive territory, but the individual results may depend on what is defined at the national and local levels. Greater focus on this level is due to the need for the practices and interventions to be evidence-based, as what is more frequently noticed is certain distance between what is suggested by the diverse evidence and the clinical practice^44^. As previously established, at this level and in this phase, the intention is to assess the individual needs, requirements arising from the pathology, family demands for daily care, skills and knowledge of the team, content of clinical treatments and also the information for patients/caregivers^41^.

*Time Dimension: Process Phase (B)*

This phase is characterized by the treatment tasks (clinical and technical) developed during provision of care. The priorities and strategies for care progress that will lead to the results of the intervention will be defined here. 

Two indicators from those previously selected were identified for the National Level, Process Phase, 1B. In general, in this phase the indicators are designated as performance/activity indicators (for example: admission rates, bed occupancy rates, mandatory treatment rates), clinical guidelines, treatment protocols and minimum care standards^33^, as can be seen in Figure 4.

**Figure 4:** Allocation of the MHIs: Process Phase

Geographical Dimension	Time Dimension
	B) Process Phase
(1) National/Regional Level	1B; 1. Students with social, emotional and mental health needs: %^*^ of these students (High School age).; 2. Hospital admission due to mental and behavioral disorders resulting from alcohol consumption: rate per 100,000 inhabitants^†^.
(2) Local Level (Take-in area)	2B; 1. Individuals hospitalized for more than 30 days in a year.; 2. Hospital readmissions due to mental illness within 30 days.; 3. Hospitalization rate per repetition year for patients with mental illness^†^.; 4. Alcohol-related hospital admission: directly standardized rate per 100,000 population^†^.; 5. Simultaneous contact with mental health and substance-abuse services due to drug abuse: %^*^ of people undergoing treatment aged over 18 years old.; 6. Students with social, emotional and mental health needs: % of these students (Elementary School age).; 7. Hospital admissions due to Mental Health problems.; 8. Mean time (duration) of acute hospitalization.; 9. Mean number of treatment days per three-month community care period.; 10. Pre-admission community service rate.; 11. Rate of hospitalization events, acute Mental Health hospital services in the public sector.
(3) Individual Level	3B; 1. Access to the services: % of people (estimated) to have anxiety/depression. People entering (in the month).

Source: Adapted^40^; ^*^% = Percentage; ^†^100,000 inhabitants = Data from the Brazilian Institute of Geography and Statistics^43^

Eleven indicators were located for the Local Level, Process Phase, 2B. For this phase, the indicators are designated for monitoring, contacts and service use patterns, audit process, paths and continuity of care and segmentation of special groups^41^.

One indicator was selected in the Individual Level, Process Phase, 3B. Here, the subjective quality of the treatments, continuity of the clinical team, frequency of appointments and the standards of the care procedures for individual patients are identified^32^. 

*Time Dimension: Results (C)*

This phase questions the evaluation of the results and should take place accurately and regularly, as a team practice, as the goal of the services is to improve the results for people with mental disorders, as well as management of the MH system in general^42^.

In the Results Phase at the National Level (1C), 8 indicators were extracted from the previously selected ones that define the efficiency and effectiveness of the interventions proposed by the service^41^. Specifically, in this phase it is possible to identify suicide rates, homeless rates (among the mentally-ill or mental illness rates among the homeless), prison rates (inadequate imprisonment of those who would be better treated in MH facilities), and special consultations (especially those in extreme adverse events such as homicides by patients). Note the indicators in the Results Phase, in Figure 5.

**Figure 5:** Allocation of the MHIs: Results Phase

Geographical Dimension	Time Dimension
	C) Results Phase
(1) National/Regional Level	1C; 1. Suicide rates - Promotion and prevention in young people.; 2. Disability complaints related to mental illness.; 3. Suicide rates - Promotion and prevention in the general population.; 4. Estimated prevalence of opiate and/or crack use: rate per 1,000 inhabitants^*^ aged from 15 to 64 years old.; 5. Excess mortality rate below the age 75 years old in adults with severe mental illness: proportion of observed and expected mortality rates (expressed in %).; 6. Gap in the employment rate for those who have contact with secondary Mental Health services and overall employment rate: percentage difference.; 7. Proportion of the population undergoing clinical treatment in Mental Health.; 8. Completed appointments.
(2) Local Level (Take-in area)	2C; 1. Prevalence of depression and anxiety in the participants (aged over 18 years old).; 2. Estimated prevalence of common Mental Health disorders (% of the population aged between 16 and 74 years old).; 3. Estimated prevalence of Mental Health disorders in children and young people (% of the population aged between 5 and 16 years old).; 4. Record of the prevalence of severe mental illness (% of the practice recorded in all age groups).; 5. Emergency hospital admissions due to intentional self-harm.; 6. Hospital admissions due to self-mutilation, standardized admission rate.; 7. Depression: prevalence recorded (individuals aged over 18 years old).; 8. Change in the consumers’ results.; 9. Post-discharge community care rate.
(3) Individual Level	3C; 1. Successful outcome of the treatment for alcoholism.

Source: Adapted^32^; *100,000 inhabitants = Data from the Brazilian Institute of Geography and Statistics^43^; ^†^% = Percentage

Nine indicators from those selected were allocated in the Local level, Results Phase, 2C. This phase directs the identification of suicide rates, aggregated results at the local level and physical morbidity^41^.

Finally, 1 indicator was defined in the Individual Level, Results Phase, 3C. The following is sought in this phase: reduction of symptoms, impacts on the caregivers, satisfaction with the services, quality of life, disabilities and needs^32^. 

The indicators selected were distributed in the different MHM dimensions, and are being made available for their use, for analysis of their viability in the clinical practice and management, as well as for scientific studies and, in the future, to be used as definers of MH policies.

## Discussion

In the results, the selection of 41 MHIs is observed through the survey with previous definitions and diverse official information, with an analysis based on EBP and properly allocated in its administrative scale, through the MHM^37^ ^-^ ^41^. 

The MHI set was allocated in the *geographical dimensions* of the Matrix (*national, local and individual levels*), as well as in the *time* dimensions (entry phase: process phase and results phase)^40^.

The MHM can be understood as a heuristic, a model, which aims at serving as a map to improve the decisions regarding care and management in MH^41^ ^,^ ^45^. Thus, one of the purposes of this MHM-based model is that it can serve as a guide in the best possible diagnosis of the condition presented by the person and in management of the MH services, so that corrective actions can be applied at the appropriate levels to improve care. The model does not claim to be rigid for prescription, but should be considered as an instrument to be used in the analysis of possible problems and in assisting the decision-making process^41^.

In order to broaden the discussion, it is worth mentioning the Donabedian Model, which provides a general structure to investigate health services and measure care quality^46^ ^-^ ^47^. It is interesting to note that, according to this model, similarly to the approach outlined in the MHM, the best way to evaluate care quality is to select a set of representative indicators from three approaches, namely: “structure”, “process” and “results”^32^. The Donabedian Model is cited here as another example of a matrix, in which the characteristics of the health practices and social organization are defined, namely: equality, coverage, user satisfaction, effectiveness, efficiency and accessibility^46^ ^-^ ^47^. In his several health research studies on health, the author did not specifically propose a model for MH; however, his work supported constitution of the domains of the Australian MHI set^39^, as well as it inspired the organization of the MHI set in England^38^.

Using the MHM to allocate the indicators presents limitations, as a heuristic needs the user’s interpretations, suggesting certain weakness in this interpretation, which may come to be applied in the continuity of these studies. Even so, this model serves to guide proper use of the MHIs, thus being able to instruct the use of these indicators in the different administrative dimensions of the Brazilian RAPS. The model focuses on assisting in the evaluation of the local services’ strengths and weaknesses and on developing an Action Plan to improve them, offering a clear approach that is also flexible enough to be relevant to their local circumstances^44^. By including the geographical dimension in the Matrix, it is perceived that the priority for organizing the MH services must be local, in order to be provided to the individuals in need. However, some of the main factors are decided regionally or nationally, based on the constant analyses of events related to the sector to define public policies and financial allocations, specific to MH^6^.

In this way, it is possible to know the aspects that can contribute to a good result for each person with an acute or chronic mental disorder episode, assisted in the primary and secondary networks. While this result is often seen as a successful achievement for the practitioners at the individual level, in the practice this includes decisions made at the local level (for example: to provide home treatment services), which must be enabled by policies and resources, decided at the national level (for example: developing community care).

The limitations found when selecting the MHIs are related to the difficulties finding diverse information with evidence of the use of indicators (and even finding these indicators with updated information) in the databases studied or in the normative documents. It was observed that it is not necessary to select a large set of indicators but, rather, to encourage a search of those that really contribute to the objectives proposed. The countries that make use of MHIs have somewhat the same indicators. 

The important thing is to obtain updated and objective data from the use of these indicators in the MH services and possible suggestions that can be adapted, aiming at improvements in the quality of the care provided to the affected population, as well as in management of the services. Another limitation to consider is non-separation of the indicators by categories, such as gender, age group, indigenous people, immigrants, street population, LGBTQIA+ and so on. However, to minimize this limitation, it is not necessary to include new indicators, only to include the variables in the implementation process of each indicator.

In agreement with the authors^40^ ^-^ ^41^, the MHM can be interpreted as a map, which serves to help establish the goals of the service and the main stages for its implementation and evaluation. However, to be adequate, this map must be simple and objective. Following this principle, the MHM was created with only two dimensions, constituting a matrix model. 

The issue of the MHIs for assisting people and managing the health network services offers a lot of room for research proposals, application in the practice and establishment of public policies^48^. Indeed, this set of indicators has application in producing evidence on a health scenario and its trends, based on experience, to identify populations with greater health needs, establish epidemiological risk and identify critical areas. In this way, it contributes as an important tool to establish policies and their priorities, to improve the quality of the services and to adapt care protocols and measures that can provide diverse information for MH promotion programs, as well as prevention and treatment of diseases with psychosocial rehabilitation of the chronic cases, seeking to better meet the needs of the population^49^ ^-^ ^51^.

Analyzing possible implications of the results of this paper, it is noted that we are only at the beginning of the research studies and proposals for the creation and organization of the MHIs aimed at the Brazilian RAPS, with all its complexity. Furthermore, the results contribute to the semantic structuring of entities and relationships found in the Mental Health Management Ontology (MHMO), an ontology for the Mental Health domain that maps clinical and biomedical aspects of this area and relates them to managerial processes of the care networks^52^ ^-^ ^53^.

This fact intensifies our interest in improving this proposal and to test it in the services, seeking consensus and accompanied by research studies and publications. As a future paper, we will submit the indicators selected in this study to the application of the Delphi Study to validate the results.

## Conclusion

This study presents a set of 41 MHIs, selected from a careful documentary analysis, seeking diverse evidence of their use in the countries selected (Evidence-Based Practice). From this selection, it is proposed that these indicators can be made available for use in the clinical practice and in management, as well as for scientific studies and, in the future, to be used as definers of MH policies. These indicators, allocated in the MHM; in the geographical dimensions: national, local and individual level; and in the time dimensions: entry phase: process phase and results phase; show possibilities and feasibility for their use in the Brazilian MH care network.

These indicators are important metrics at all RAPS levels and services, acting to show the conditions of management, incidence, prevalence, mortality and morbidity, allowing through their information that managers can intervene in the improvement of the entire network of services and even anticipate promotion and prevention measures in MH. 

Due to non-use of official MHIs in Brazil, it is expected that the results herein presented will be a stimulus for new research studies, as well as an aid for establishing policies aimed at the priorities, specific laws, quality of the services, adaptation of care protocols and measures that can provide diverse information to the MH programs, always seeking to better meet the needs of the population.
